# Comparative study of myocardial perfusion and coronary flow velocity reserve derived from adenosine triphosphate stress myocardial contrast echocardiography in coronary lesions with no/mild stenosis

**DOI:** 10.3389/fcvm.2024.1353736

**Published:** 2024-09-23

**Authors:** Xuebing Liu, Chunmei Li, Qingfeng Zhang, Qingguo Meng, Hongmei Zhang, Zhaohuan Li

**Affiliations:** ^1^ Department of Cardiovascular Ultrasound and Non-Invasive Cardiology, Sichuan Provincial People’s Hospital, School of Medicine, University of Electronic Science and Technology of China, Chengdu, China; ^2^ Ultrasound in Cardiac Electrophysiology and Biomechanics Key Laboratory of Sichuan Province, Sichuan Provincial People’s Hospital, School of Medicine, University of Electronic Science and Technology of China, Chengdu, China

**Keywords:** adenosine triphosphate stress, myocardial contrast echocardiography, myocardial perfusion, coronary flow velocity reserve, coronary microvascular disease

## Abstract

**Background:**

Qualitative myocardial perfusion (QMP) derived from myocardial contrast echocardiography reflects the capillary flow, while coronary flow velocity reserve from Doppler spectrum (D-CFVR) of the left anterior descending coronary artery (LAD) is used to assess coronary microvascular function, particularly after excluding severe epicardial coronary stenosis. The present study aimed to assess the relationship of QMP and D-CFVR in detecting coronary microvascular disease (CMVD) by using adenosine triphosphate stress myocardial contrast echocardiography (ATP stress MCE).

**Methods and results:**

Seventy-two patients (mean age: 54.22 ± 12.78 years) with chest pain and <50% coronary stenosis diagnosed by quantitative coronary angiography or dual-source CT underwent ATP stress MCE. The distribution of myocardial perfusion and CFVR value was estimated by experienced physicians. Of the 72 LAD with 0%–50% diameter stenosis, 15 (21%) exhibited abnormal CFVR and 31 (43%) displayed abnormal perfusion with ATP stress MCE. Eleven of the 15 LAD territories (73%) with abnormal CFVR values showed abnormal perfusion. However, CFVR was considered normal in 20 LAD territories (35%), despite the presence of perfusion defect in the territory.

**Conclusion:**

Abnormal myocardial perfusion during ATP stress MCE was found in a sizable percentage of patients in whom CFVR of the supplying vessel was considered normal.

## 1 Introduction

The subset of disorders affecting the coronary microcirculation is termed coronary microvascular disease (CMVD), which is characterized by reduced coronary flow reserve (CFR), increased coronary microvascular resistance, microvascular spasm, coronary endothelial dysfunction, and microembolism (1). Wang et al. and Zhang et al. reported that 70% of patients with angina undergoing coronary angiography did not exhibit coronary obstruction, and the major reason for this might be related to CMVD (2, 3). Presently, the gold standard to diagnose CMVD is the invasive coronary reactivity test, which is hardly accepted by most patients. According to the 2020 European Consensus of Experts on Non-Obstructive Coronary Ischemia, non-invasive examination methods are recommended as the preferred diagnostic process for patients with angina pectoris (4). If dual-source computed tomography (CT) does not detect obstructive coronary heart disease, or functional tests do not detect reversible segmental myocardial ischemia, subsequent non-invasive or invasive examination methods should be used to determine whether the patient has coronary artery microcirculation disorders or vasospasm angina. Common non-invasive methods for evaluating microcirculation disorders include single-photon emission computed tomography (SPECT), stress CT perfusion, transthoracic coronary artery flow imaging, myocardial contrast-enhanced echocardiography, positron emission computed tomography (PET) and cardiac magnetic resonance imaging (CMRI). SPECT, stress CT perfusion and PET involve the risk of radiation, while CMRI is constrained in patients with metal implants. Moreover, iodine-based contrast agents have a potential nephrotoxic effect. Regarding the gadolinium-based contrast media (GBCM) used for CMRI, it is considered to have a low risk of nephrotoxicity or other side effects. However, some reports have demonstrated the potential toxicity of GBCM, especially in patients with kidney impairment. Finally, all three modalities are costly.

Microbubble is popularly used as an ultrasonic contrast agent because it is smaller than red blood cells and can pass through myocardial microvessels. Comparative analysis of myocardial contrast-enhanced imaging is used to rapidly and conveniently detect myocardial perfusion. Some studies have shown that quantitative myocardial perfusion derived from adenosine triphosphate stress myocardial contrast echocardiography (ATP stress MCE) can assess CMVD accurately and provide considerable prognostic value (5, 6). However, quantitative stress MCE requires special software and a long processing time, which could be limiting factors for the actual use of this technique in clinical settings. Qualitative analysis of stress MCE can conveniently detect abnormal myocardial perfusion (7).

Coronary flow velocity reserve from transthoracic Doppler echocardiography (D-CFVR) is a simple and economical method, and it can be used to reflect the reserve function of coronary microvessels (8, 9). D-CFVR is mainly estimated by measuring the ratio of the maximum diastolic blood flow and resting blood flow in the distal segment of the left anterior descending coronary artery (LAD). In the absence of epicardial coronary occlusion, the reduction in CFVR can reflect CMVD and is the independent predictor of adverse events (10, 11). The distal coronary blood flow spectrum in patients with acute anterior myocardial infarction can also predict myocardial microvascular injury (12).

A direct comparison of qualitative myocardial perfusion (QMP) and D-CFVR is currently lacking, particularly using ATP stress MCE in the setting of no/mild coronary stenosis. Therefore, in the present study, we assessed the relationship of QMP and D-CFVR derived from ATP stress MCE in detecting CMVD in coronary lesions with no/mild stenosis.

## 2 Methods

### 2.1 Study population

**Figure 1:**
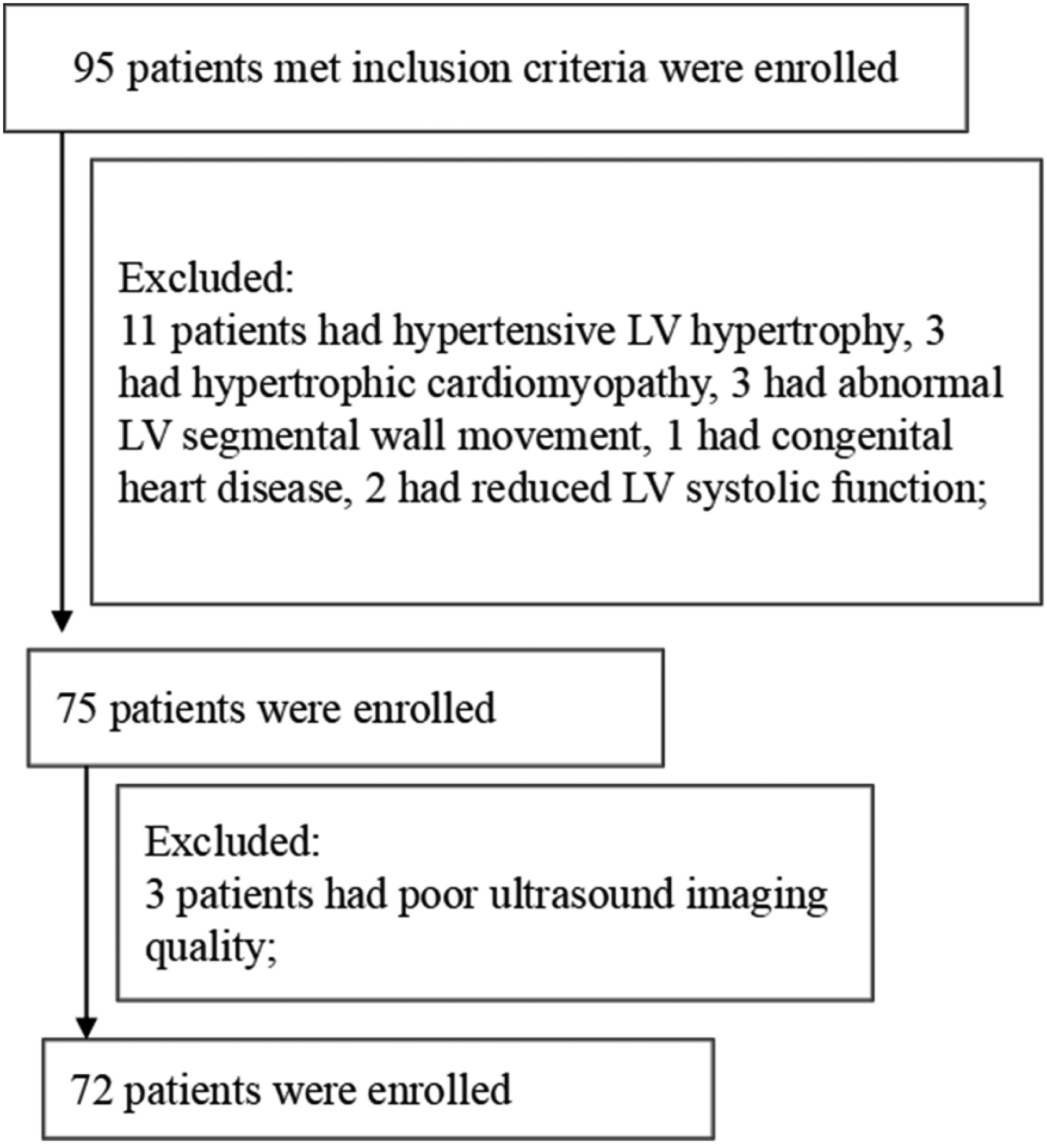
Flowchart describing the patient selection process.

Ninety-five patients with chest pain and chest tightness who underwent ATP stress MCE from July 2020 to November 2021 were retrospectively analyzed. The inclusion criteria were as follows: patients showing <50% stenosis of the left and right coronary arteries and branches in coronary angiography or dual-source CT. After excluding patients with segmental wall motion abnormalities of the left ventricle (LV) at rest, LV hypertrophy, congenital heart disease, reduced LV systolic function, prior myocardial infarction, prior coronary artery reconstruction (coronary artery bypass or stenting), severe valvular disease, asthma, atrioventricular block, hypersensitivity, myocardial bridge and poor ultrasound imaging quality, we finally enrolled 72 patients (Figure 1). This study was approved by the Ethics Committee of Sichuan Provincial People's Hospital, and signed informed consent was obtained from all patients. The present study was performed in accordance with the relevant guidelines and regulations.

### 2.2 Routine echocardiography

Images were obtained using a GE Vivid E9 color Doppler ultrasound device (GE Inc., Boston, USA) equipped with a phased array probe M5S (1.5–4.6 MHz) (GE Inc.). Images were acquired according to the protocol of the American Society of Echocardiography.

### 2.3 CFVR and QMP protocol

**Figure 2:**
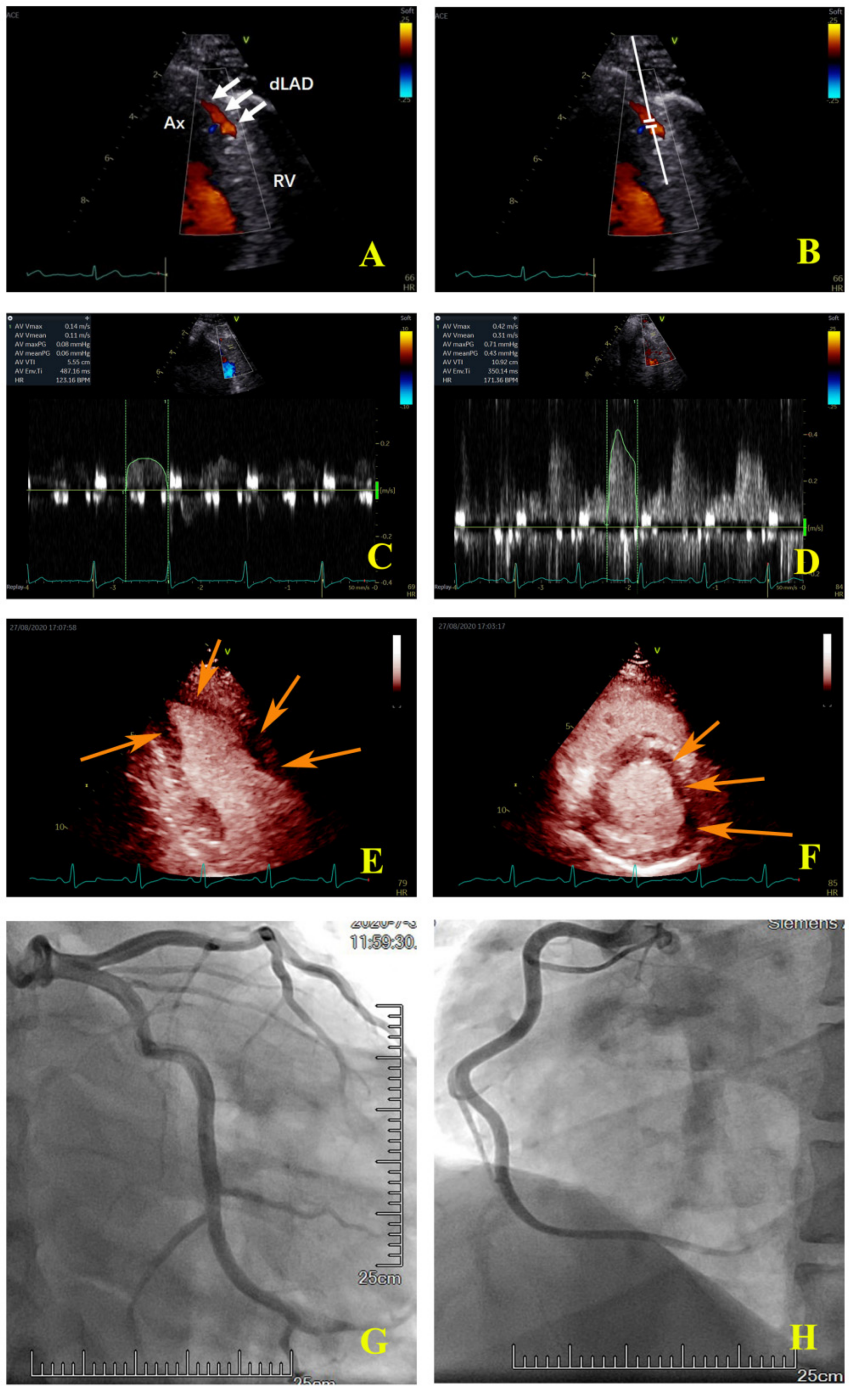
Coronary colour Doppler and blood flow spectrum and MCE and angiographic images of the same patient. **(A)** The colour Doppler in the distal left anterior descending artery (white arrow); **(B)** the sample volume was placed in the distal left anterior descending artery; **(C)** the coronary flow velocity in the distal left anterior descending artery at rest; **(D)** the coronary flow velocity in the distal left anterior descending artery at peak after stress, CFVR = 2.81; **(E)** the apical two-chamber view showed that the myocardial perfusion of LV apical and anterior wall was sparse (orange arrow); **(F)** the middle section of the LV short axis view showed that the myocardial perfusion of the anterior wall and anterior septum was sparse (orange arrow); **(G)** the left coronary artery angiography. **(H)** the right coronary artery angiography. Ax, apex; RV, right ventricle; dLAD, the distal left anterior descending artery.

The ultrasound machine was switched into the coronary Doppler mode at 1.9–4.0 MHz auto frequency. The color Doppler scale was set at 15 cm/s with minor adjustment in apecial cases. Keeping the frame rate not less than 25 Hz, the color box size was adjusted to ensure the clarity of the image and reduce the artifacts. The depth was set at 8–12 cm. The flow of the distal LAD was visualized in the modified apical three-chamber view by slightly adjusting the probe (Figure 2A). The sample volume was placed in the color Doppler area of the LAD (Figure 2B). The coronary blood flow spectrum of the LAD was obtained at rest. The probe was maintained at the same site throughout the image acquisition process. ATP (140 µg/min/kg) was pumped through the cephalic vein of the forearm for approximately 6 min. At the third minute after ATP infusion, the coronary blood flow spectrum of the LAD was again obtained. The ultrasound machine was then switched into the MCE mode and the SonoVue solution was injected into the cephalic vein at an even speed (1 ml/10 s). The Sono Vue solution was prepared as follows: First, 59 mg of SonoVue (Bracco Suisse SA, Switzerland) was dissolved in 5.0 ml of normal saline and shaken until the powder was completely dispersed; next, 1.5 ml of the solution was extracted each time, diluted in 5.0 ml of normal saline. After the left ventricular myocardium was filled with a sufficient amount of the contrast agent, a high-energy pulse was triggered to destroy the contrast microbubbles in the myocardium, and the pulse was then automatically switched to a low-energy (mechanical index = 0.1) real-time contrast mode. Cine images of the apical four-, three-, and two-chamber views and the middle section of the LV short axis view were collected for playback and offline analysis. Each cine included 10 cardiac cycles after the trigger of a high energy pulse.

### 2.4 Image and data analysis

Left ventricular ejection fraction (LVEF) was measured using the biplane Simpson's method.

Two experienced physicians blinded to angiographic/CT and CFVR data independently analyzed the myocardial perfusion of each patient. In the case of disagreements, the third physician was consulted, and a consensus was reached. Abnormal perfusion was defined as the myocardial perfusion that did not reach the peak until 2 s after a high mechanical index impulse during stress; this showed that the area of myocardial perfusion was significantly delayed and sparse compared to the surrounding area (9, 13, 14). Then the determination of the coronary supply territories (LAD/LCX/RCA territories) with abnormal myocardial perfusion based on coronary anatomy at coronary angiography or CT.

The diastolic maximum and mean flow velocities of the LAD were measured at rest (Rest-V_max_ and Rest-V_mean_, respectively) and at peak (Peak-V_max_ and Peak-V_mean_, respectively) based on the Doppler spectrum. Each parameter was analyzed at the interval of three heartbeats, and the average value was obtained. CFVR was calculated according to the formula: CFVR = Peak-V_mean_/Rest-V_mean_. An abnormal CFVR was defined as a value of less than 2 (15, 16).

### 2.5 Repeatability of measurements

In all, the myocardial contrast images of 20 subjects were randomly selected, and their myocardial perfusion were independently analyzed by Li Z (12 years of work experience) and Liu X (more than 10 year of work experience). Two weeks later, Liu X reanalyzed the images.

### 2.6 Statistical analysis

Data are expressed as mean ± standard deviation (SD). The Kolmogorov-Smirnov method was used to test the normality of the variance. The homogeneity of variance was detected by the Levene method. Student's *t*-test or Mann-Whitney test was used for comparing two groups. Multiple groups were compared with One-way ANOVA and the comparison among groups were performed with SNK test. Categorical data were compared by the chi-square test. Inter-observer and intra-observer consistency tests were performed by intra-class correlation coefficient (ICC). A two-tailed *p*-value of <0.05 was considered statistically significant. Statistical data were analyzed by IBM SPSS 19.0 (IBM Inc., New York, USA).

## 3 Results

The study enrolled 72 patients, including 35 men and 37 women. The age of the patients ranged from 22 to 79 years, and the mean age was 54.22 ± 12.78 years. Among the 72 patients, 59 patients underwent coronary angiography, and the remaining 13 patients received dual-source CT. Table 1 shows the general characteristics of the patients.

**Table 1:** Characteristics of the study patients.

	Total patients (*n* = 72)
Age, years	54.22 ± 12.78
Sex, male	35 (48.6%)
SBP, mmHg	118 ± 18
DBP, mmHg	70 ± 13
HR, bpm	85 ± 14
HTN	27 (37.5%)
Hyperlipidemia	22 (30.5%)
Diabetes mellitus	18 (25.0%)
Obesity	8 (11.1%)
Chronic nephritis	2 (2.8%)
LVEF	0.69 ± 0.04
HFpEF	19 (26.4%)
Smoker	16 (22.2%)
Statins	29 (40.3%)
Antidiabetic agents	7 (9.7%)
β blockers	23 (31.9%)
Aspirin or clopidogrel	17 (23.6%)
Calcium channel blockers	20 (27.8%)
Nitrates	7 (9.7%)
ACEI or ARBs	21 (29.2%)

Data are expressed as mean ± SD for continuous variables and *n* (%) for categorical variables.

SBP, systolic blood pressure; DBP, diastolic blood pressure; HTN, hypertension; LVEF, left ventricular ejection fraction; HFpEF, heart failure with preserved ejection fraction; ACEI, angiotensin-converting-enzyme inhibitor; ARB, angiotensin II receptor blockers.

### 3.1 Safety of the examination

All the patients tolerated well the entire examination process. Forty-four patients showed only mild symptoms such as palpitations. Twenty-four patients showed abnormal results in electrocardiography, with low and flat or inverted T wave and depressed ST segment. During the stress, 3 patients had atrial premature beats, 3 patients had ventricular premature beats, 1 patient had first-degree atrioventricular block, and 1 patient had second-degree atrioventricular block type 1; all these patients recovered after withdrawal of the stress.

### 3.2 Detection of CMVD by QMP

Forty-three patients were detected abnormal QMP, among which the LAD territories were involved in 26 patients, the left circumflex artery (LCX) territories were involved in eight patients, the right coronary artery (RCA) territories were involved in four patients, both LAD and RCA territories were involved in three patients, and the three coronary artery territories were involved in two patients (Table 2). Of the 216 vessels with 0%–50% diameter stenosis, 50 (23%) vessels exhibited abnormal perfusion in the coronary artery territory with ATP stress MCE, in which 31 (62%) located in the LAD territories (Tables 2, 3). The proportions of patients with abnormal QMP were almost the same in the subgroups with different stenosis of coronary arteries (Table 4).

**Table 2:** Results of detection based on qualitative myocardial perfusion and coronary flow velocity reserve.

	Patients (Territories)
Qualitative myocardial perfusion	
Total	72 (216)
Abnormal	43 (50)
Normal	29 (166)
Location of abnormal territories	
Total	43 (50)
Only LAD	26 (26)
Only RCA	4 (4)
Only LCX	8 (8)
LAD + RCA	3 (6)
LAD + RCA + LCX	2 (6)
CFVR	
Total	72 (72)
Abnormal	15 (15)
Normal	57 (57)

CFVR, coronary flow velocity reserve; LAD, left anterior descending coronary artery; LCX, left circumflex coronary artery; RCA, right coronary artery.

**Table 3:** Diagnostic results of qualitative myocardial perfusion and coronary flow velocity reserve for CMVD.

Technology	Diagnostic results	QMP [Patients (LAD territories)]		
		Positive	Negative	Total
CFVR [Patients (LAD territories)]	Positive	11 (11)	4 (4)	15 (15)
	Negative	32 (20)	25 (37)	57 (57)
	Total	43 (31)	29 (41)	72 (72)

CFVR, coronary flow velocity reserve; QMP, qualitative myocardial perfusion.

**Table 4:** Comparison of subgroups with different stenosis of coronary arteries.

	CFVR value	CFVR <2.0	Abnormal QMP
20%–50% coronary stenosis (*n* = 18)	2.76 ± 1.11	7 (38.9%)	14 (77.8%)
<20% coronary stenosis (*n* = 39)	3.03 ± 0.76	5 (12.8%)	21 (53.8%)
Normal coronary artery (*n* = 15)	2.75 ± 0.73	3 (20.0%)	8 (53.3%)
Inspection value	*F* = 0.94	χ^2^ = 5.08	χ^2^ = 3.25
*P*-value	*P* = 0.39	*P* = 0.08	*P* = 0.20

Data are expressed as mean ± SD for continuous variables and *n* (%) for categorical variables.

CFVR, coronary flow velocity reserve; QMP, qualitative myocardial perfusion.

### 3.3 Detection of CMVD by D-CFVR

Fifteen patients were detected abnormal D-CFVR, and all of them showed the involvement of the LAD territories. Of the 72 LAD with 0%–50% diameter stenosis, 15 (21%) exhibited abnormal CFVR with ATP stress (Table 2). The values of CFVR and the proportions of patients with negative CFVR was almost the same in the subgroups with different stenosis of coronary arteries (Table 4).

### 3.4 QMP vs. CFVR

**Figure 3:**
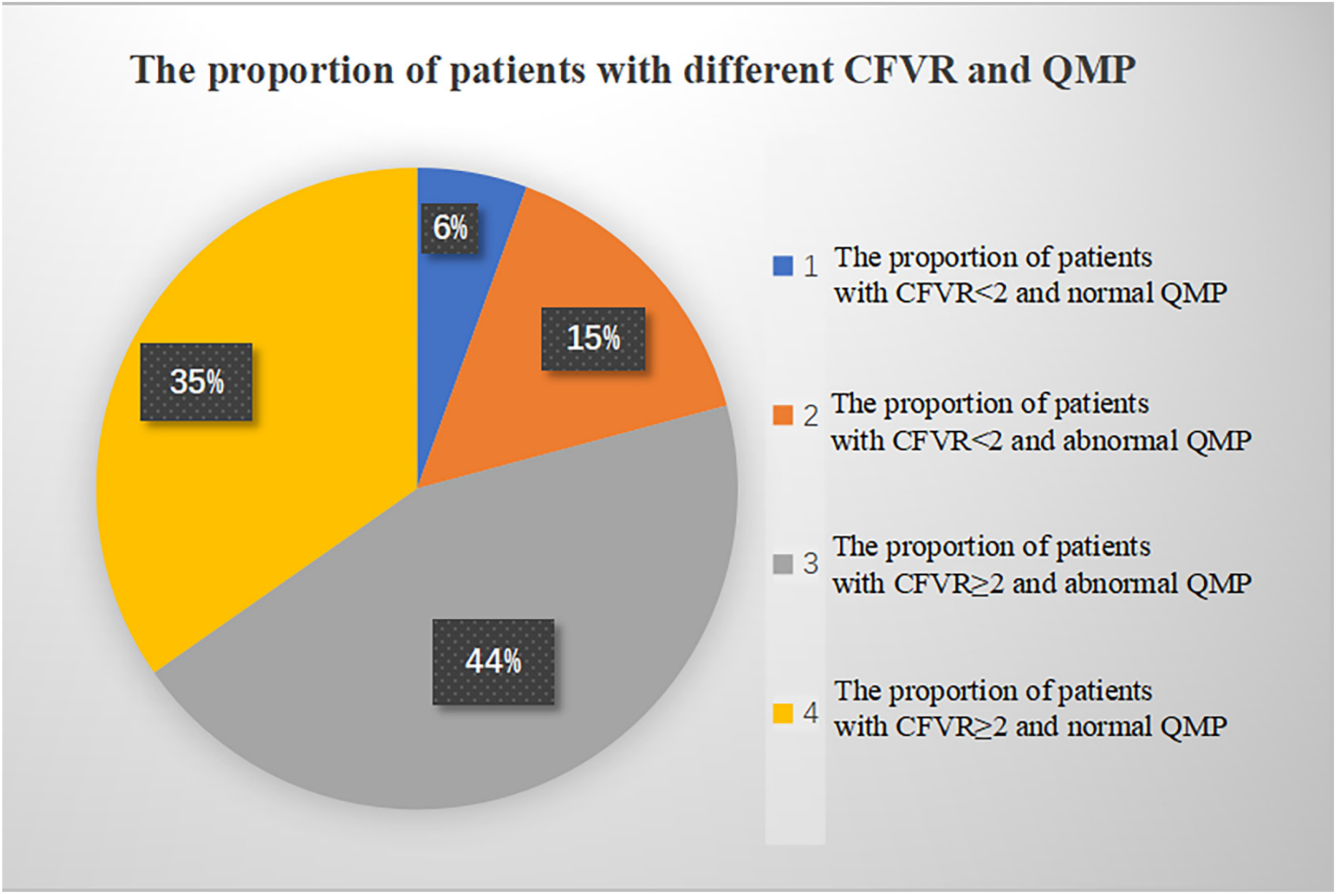
The proportion of patients with different CFVR and QMP. CFVR, coronary flow velocity reserve; QMP, qualitative myocardial perfusion.

CMVD was detected in 47 patients by QMP and/or CFVR, among which 43 patients were positive in QMP, 15 in CFVR, and 11 in both, while 29 patients were negative in QMP, 57 in CFVR, and 25 in both (Table 3). Significant differences were observed in the proportion of times between an abnormal CFVR study and an abnormal QMP study and between a normal CFVR study and a normal QMP study (*P* = 0.000, Fisher's exact test). The proportion of patients with different CFVR and QMP is shown in Figure 3.

Eleven of the 15 LAD territories (73%) with abnormal CFVR values had abnormal perfusion (Table 3). However, in 20 LAD territories (35%), CFVR was considered normal despite the presence of an induced perfusion defect in the territory (Table 3) (examples are shown in Figure 2 for the LAD territories). In the CFVR vessels with values >2.00, no difference in CFVR was observed for those with abnormal QMP in the LAD territory vs. normal perfusion studies; however, a significant decrease was noted in Rest-V_mean_, Peak-V_mean_, Rest-V_max_, and Peak-V_max_ values (Rest-V_mean_: 0.16 ± 0.04 m/s vs. 0.18 ± 0.05 m/s, Peak-V_mean_: 0.49 ± 0.14 m/s vs. 0.57 ± 0.15 m/s, Rest-V_max_: 0.21 ± 0.07 m/s vs. 0.25 ± 0.05 m/s, Peak-V_max_: 0.71 ± 0.21 m/s vs. 0.83 ± 0.19 m/s; all *p* < 0.05) (Table 5).

**Table 5:** Comparison of the coronary flow velocity between the abnormal and normal perfusion LAD territories in normal CFVR territories.

	CFVR	Rest-V_mean_ (m/s)	Peak-V_mean_ (m/s)	Rest-V_max_ (m/s)	Peak-V_max_ (m/s)
Abnormal perfusion group (*n* = 20)	3.20 ± 0.93	0.16 ± 0.04	0.49 ± 0.14	0.21 ± 0.07	0.71 ± 0.21
Normal perfusion group (*n* = 37)	3.18 ± 0.61	0.18 ± 0.05	0.57 ± 0.15	0.25 ± 0.05	0.83 ± 0.19
*t*	−0.11	2.33	2.16	2.63	2.24
*P*	0.91	0.02	0.04	0.01	0.03

Data are expressed as mean ± SD.

CFVR, coronary flow velocity reserve; Rest-V_mean_, the mean flow velocity of the distal LAD at rest; Peak-V_mean_, the mean flow velocity of the distal LAD at peak after stress; Rest-V_max_, the maximum flow velocity of the distal LAD at rest; Peak-V_max_, the maximum flow velocity of the distal LAD at peak after stress.

### 3.5 Repeatability of measurements

The inter-observer and intra-observer ICCs values of QMP were 0.890 and 0.943, respectively (*P* < 0.001), indicating good reproducibility and reliability of the tests.

### 3.6 Prognosis

Among the 32 patients who detected abnormal QMP but negative CFVR, in addition to the treatment of basic diseases such as hypertension, hyperlipidemia and diabetes, after antiplatelet and vasodilator therapy for 1–6 months (median duration of follow-up, 3 months), 28/32 (87.5%) patients had significant relief of chest pain and chest tightness. Four patients still had episodes of chest pain and chest tightness, but the interval between attacks is longer than before.

## 4 Discussion

Presently, CMVD is attracting increasing attention of clinicians, and the noninvasive detection of CMVD has become a research hotspot in recent years. In the present study, we observed that ATP is an ideal vasodilator for stress echocardiography. QMP during ATP stress MCE has a high sensitivity for detecting coronary microvascular dysfunction in a sizable percentage of patients in whom CFVR of the supplying vessel is considered normal. This might be because of lower coronary microvascular flow velocity in these patients and the location of some lesions in vessels that were not supplied by the LAD. Although there is a high variability in the rate of CMVD diagnosis based on the current methods used which is influenced by multiple factors (17), the reproducibility and reliability of the QMP can be acceptable according to the ICCs.

Poor acoustic window and the operator's training level are recognized as potential limitations of stress echocardiography in clinical practice. Nevertheless, stress echocardiography remains one of the most cost-effective imaging modalities, as it does not involve radiation exposure and demonstrates good accuracy for detecting anatomically significant CAD. According to Knuuti et al. (18), stress echocardiography has a sensitivity of 85% (range: 80%–89%) and a specificity of 82% (range: 72%–89%). ATP was selected as the vasodilator in the present study. Although the vasodilator effect of ATP is similar to that of adenosine, ATP has a better price advantage and a longer half-life which is enough to simultaneously evaluate both CFVR and QMP (5). In this study, 60% of the patients experienced different symptoms during the stress period; however, they were able to endure the entire trial, and their symptoms were quickly relieved after ATP stress was withdrawn.

In this study population, all symptomatic patients showed no evidence of anatomically significant (>50%) coronary artery disease. Because most studies on coronary microvascular lesions detected by ATP stress MCE have focused on the regional segments, no apparent change is noted in left ventricular systolic function; consequently, the severity of the disease tends to be underestimated in the clinic.

In the present study, QMP detected coronary microvascular dysfunction to a greater extent than CFVR. ATP stress MCE can directly show the perfusion of coronary microvessels. Perfusion delay and/or a defect in coronary microcirculation may manifest as the presence of sclerosis, stenosis, microembolism, spasm, and compression of the coronary microvessels (11, 12). D-CFVR can assess the vasodilatory capacity of the LAD. Although most coronary microvascular lesions are concentrated in the apex (12), D-CFVR fails to assess the lesions located in vessels that are not supplied by the LAD. These undetectable areas for D-CFVR can be evaluated by QMP. As observed in the present study, 19 vessel territories with abnormal QMP were not located in the LAD territories. On the other hand, in the normal CFVR studies, the abnormal QMP territories were associated with lower values of Rest-V_mean_, Peak-V_mean_, Rest-V_max_, and Peak-V_max_; this implied that the CFVR(-) group with an abnormal QMP had a slower coronary flow state during the static state and demand stress.

Coronary slow flow (CSF) is associated with elevated microvascular resistance, and it is recognized as evidence of impaired microvascular function, commensurate with a diagnosis of CMVD (14, 19–21). The risk factors of CSF are mainly related to male sex, smoking, hyperlipidemia, anxiety, and depression. Chest pain and tightness are the common symptoms of CSF (22, 23). In our present study, QMP revealed myocardial perfusion delay in patients with CSF, while the CFVR value remained normal. Fineschi et al. conducted a small study of 15 patients by using the intracoronary thermodilution method and observed that patients with CSF had elevated resting microvascular resistance but normal CFVR values (24).

Utkarsh et al. used a CFR value of <2.5 to define coronary microvascular dysfunction (16); they found that CSF had poor sensitivity and specificity for coronary microvascular dysfunction (26.7% and 65.2%, respectively), which indicated that CSF should not be considered synonymous with coronary microvascular dysfunction definded according to CFVR (25). The mechanism of CMVD is related to abnormal coronary microvascular structure and function (26). QMP, as a standalone index reflecting myocardial blood flow, and CFVR defined by the ratio of resting to hyperemic flow velocity, could be used to assess CMVD of different etiologies. CFVR can reflect the relaxation function of resistive coronary arteries in response to pharmacological and physiological interventions. In addition to abnormal relaxation function, CSF can also occur due to several structural changes, such as microvascular remodeling and microembolism, which might not be associated with the abnormal dilation function of microvessels. A very recent study revealed hidden coronary epicardial atherosclerosis as assessed by intravascular ultrasounds and not a low CFVR as assessed by transthoracic Doppler has been found as the underlying abnormalities in the patients with CSF (27). Compared to CFVR, QMP seems to be superior in detecting CSF caused by structural alterations in coronary microvascular functions. The prognosis shows most patients with abnormal QMP but negative CFVR relieved after antiplatelet and vasodilator therapy. Hence, we think that CFVR cannot be used to detect all types of CMVD.

A diffuse non-obstructive coronary stenosis is one of the reason of low CFVR and high baseline flow velocity (28). In the study, 15 patients with abnormal CFR might resulted from diffused coronary atherosclerosis or CMVD. The coronary angiography images of these 15 patients showed that 8 of them had no coronary stenosis, 2 had non-LAD stenosis, and 5 had <50% LAD stenosis. One of the 5 patients with mild LAD stenosis were diffuse lesions. Therefore, the reduction of CFR in 14 of the 15 patients was considered to be related to CMVD, and that in the other one cases was possibly caused by diffuse coronary lesions. The transthoracic Doppler as reported has a potential value in assessing diffuse coronary artery disease, and such an approach has got now very high feasibility in flow mapping the entire LAD (29). CFVR combined with baseline flow velocities in the whole LAD by transthoracic coronary Doppler ultrasound can noninvasively distinguish CMVD from diffuse coronary lesions.

## 5 Study limitations

This study was a single-center retrospective study, and selection bias may have influenced the results; hence, prospective studies are required to test the correlation of QMP with CFVR in no/mild stenosis and the relevance of QMP combined with CFVR based on ATP stress MCE in cardiovascular risk prediction for patients with CMVD. All the enrolled patients did not receive the invasive coronary reactivity test to detect CMVD; hence, there was no gold standard to validate the accuracy of the two noninvasive methods. However, the primary purpose of our study was to evaluate the differences and relationships between QMP and CFVR in detecting CVMD in vessels exhibiting 0%–50% diameter stenosis. Our study mirrored real-world clinical settings where QMP and CFVR are used to evaluate and guide the management of lesions with no/mild stenosis. The myocardial perfusion deficit may also reflect a functionally significant non-obstructive (≤50%) epicardial stenosis, which was not ruled out in this study. CFVR cannot distinguish CMVD from diffuse non-obstructive coronary stenosis. Finally, the small sample size limited our ability to perform deeper subgroup analysis. Longitudinal studies with larger sample sizes and longer follow-up periods are needed to validate the prognostic significance of qualitative and quantitative analyses obtained by echocardiography in patients with suspected CMVD.

## 6 Conclusions

The findings of the present study confirmed that abnormal QMP with ATP stress MCE had a high sensitivity for detecting an abnormal CFVR in a vessel territory with no/mild stenosis; however, an abnormal CFVR value had a low sensitivity in predicting abnormal QMP. This might result from a slow coronary microcirculation during the static state and demand stress, which is a critical pathophysiological mechanism of abnormal myocardial perfusion in patients with no/mild epicardial stenosis.

Our present study demonstrated that relying on CFVR alone in evaluating lesions with no/mild stenosis may require further consideration if the territories exhibit lower coronary microvessel blood flow during the static state and demand stress.

## Data Availability

The original contributions presented in the study are included in the article/Supplementary Material, further inquiries can be directed to the corresponding author.
